# Alarming Proportions of Methicillin-Resistant *Staphylococcus aureus* (MRSA) in Wound Samples from Companion Animals, Germany 2010–2012

**DOI:** 10.1371/journal.pone.0085656

**Published:** 2014-01-20

**Authors:** Szilvia Vincze, Ivonne Stamm, Peter A. Kopp, Julia Hermes, Cornelia Adlhoch, Torsten Semmler, Lothar H. Wieler, Antina Lübke-Becker, Birgit Walther

**Affiliations:** 1 Institute of Microbiology and Epizootics, Veterinary Faculty, Free University Berlin, Germany; 2 Vet Med Labor GmbH, Division of IDEXX Laboratories, Ludwigsburg, Germany; 3 Department of Infectious Diseases Epidemiology, Robert Koch Institute, Berlin, Germany; Columbia University, College of Physicians and Surgeons, United States of America

## Abstract

*Staphylococcus* (*S*.) *aureus* is an important cause of wound infections in companion animals, and infections with methicillin-resistant *S. aureus* (MRSA) are of particular concern due to limited treatment options and their zoonotic potential. However, comparable epidemiological data on MRSA infections in dogs, cats and horses is scarce, also limiting the knowledge about possible links to MRSA isolates from human populations. To gain more knowledge about the occurrence and genotypic variation of MRSA among wound swabs of companion animal origin in Germany we performed a survey (2010–2012) including 5,229 samples from 1,170 veterinary practices. *S. aureus* was identified in 201 (5.8%) canine, 140 (12.2%) feline and 138 (22.8%) equine swabs from a total of 3,479 canine, 1,146 feline and 604 equine wounds, respectively. High MRSA rates were identified with 62.7%, 46.4% and 41.3% in *S. aureus* of canine, feline and equine origin, respectively. Further genotyping including *spa* typing and multilocus sequence typing (MLST) revealed a comparable distribution of *spa* types among canine and feline MRSA with CC22 (47.6%; 49.2%) and CC5 (30.2%; 29.2%) as predominant lineages followed by CC398 (13.5%; 7.7%) and CC8 (4.0%; 9.2%). In contrast, the majority of equine MRSA belonged to CC398 (87.7%). Our data highlight the importance of *S. aureus* and MRSA as a cause of wound infections, particularly in cats and horses in Germany. While “human-associated” MRSA lineages were most common in dogs and cats, a remarkable number of CC398-MRSA was detected in horses, indicating a replacement of CC8-MRSA as the predominant lineage within horses in Germany. These data enforce further longitudinal epidemiological approaches to examine the diversity and temporal relatedness of MRSA populations in humans and animals to assess probable sources of MRSA infections. This would enable a sound risk assessment and establishment of intervention strategies to limit the additional spread of MRSA.

## Introduction

*Staphylococcus (S.) aureus* is an important pathogen in human and veterinary medicine that is capable of causing purulent and toxin-mediated infections. An uptake of the *mec*A gene, which is encoded on the Staphylococcal cassette chromosome *mec*, enables the expression of an additional penicillin-binding protein (PBP)2a which substitutes an essential cross-linking step of the native PBP2 in the presence of ß-lactams, leading to resistance against antibiotics of this group [1]. In addition, methicillin-resistant *S. aureus* (MRSA) are often resistant against further antimicrobial classes [2].

MRSA raised attention in companion animal medicine during the last decades, especially with regard to transmission from humans to dogs and cats and vice versa [3]–[5]. Further, MRSA are known as important nosocomial pathogens in both human and veterinary medicine [6].

Data concerning the proportion and impact of *S. aureus* and MRSA infections in clinical samples of companion animals are variable but give evidence of an increased infection rate in horses [7] as well as dogs and cats [8] during the last years. Wound infections in particular are described to be frequently caused by *S. aureus* [9], [10].

Genotypic characterization of MRSA from several infection sites of dogs and cats revealed the predominant occurrence of well-known regional hospital associated (HA-) MRSA [5], [11]–[13]. Within Germany, clonal complex (CC) 22-MRSA was reported as the most common lineage in small animals in 2003 and 2004 [9], [13]. Only recently, CC398-MRSA was reported as a cause of canine infections in Germany, as well [14], [15].

In contrast, CC8-MRSA were mainly identified in equine infections, leading to suggestions of adaptation of this lineage to horses [16]. In the recent past, studies revealed the occurrence of LA-MRSA CC398 in horses, as well [7], [16]–[18].

Suspected transmission events between humans and small animals or horses were reported several times in the past [3]–[5], [19] and strains of these lineages with the ability to infect different hosts were denominated as Extended Host Spectrum Genotypes (EHSGs) [17].

The predominance of infections with regularly occurring regional human lineages in companion animals raised the question whether dogs and cats might be an important source of human infection and colonization [20]. Therefore, continuous *S. aureus-*screening in specimens of companion animal origin with further molecular characterization is important to determine changes in frequency or possible adaptation of specific lineages to different hosts.

Hence, we conducted a Germany-wide survey to investigate the frequency of *S. aureus* among samples from clinical wound infections of dog, cat and horse origin. The MRSA-proportion was identified for *S. aureus*-positive samples. Each phenotypically methicillin-resistant *S. aureus* was verified by PCR detection of either the *mec*A or *mec*C gene and characterised by *spa* typing as well as screening for the Panton-Valentine leukocidin (PVL).

## Materials and Methods

### Sampling and Case Definition

**Figure 1:**
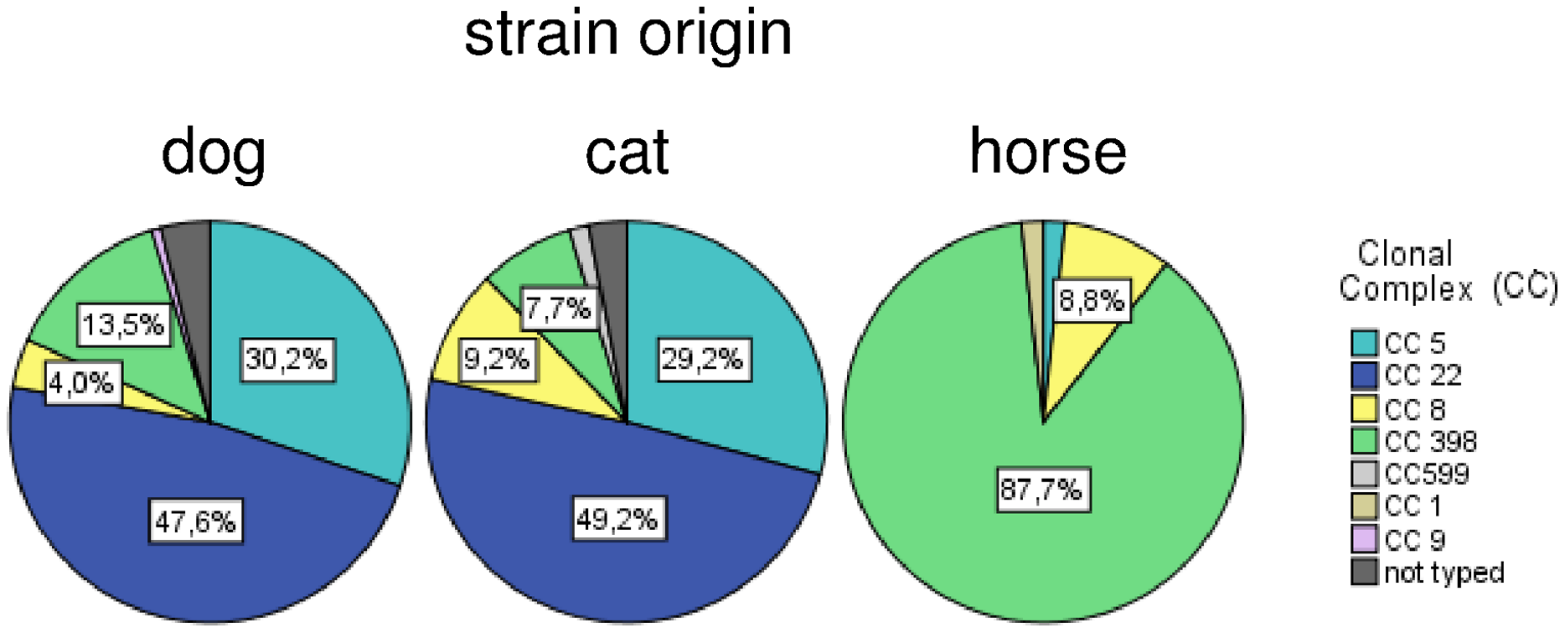
Sample origin. Figure 1 shows the Germany-wide origin of the 5,229 wound swabs from dogs, cats and horses. Areas are shaped in color with regard to the sample frequency. Black dots represent the sample origin with regard to the postal code. The dot size displays the submission frequency of each veterinary practice/clinic.

We included all swabs from dogs, cats and horses suffering from wound infections, which were sent from 1,170 veterinarians within Germany to Vet Med Labor GmbH (Ludwigsburg, Germany) for bacteriological analysis between November 2010 and March 2012. Vet Med Labor GmbH is one of the largest veterinary microbiological laboratories, covering most areas within Germany. The samples originated from either veterinarian clinicians or practitioners working at mixed or specialized practices in 1,018 different areas with regard to the postal code throughout Germany. TargetMap (www.targetmap.com) was used to visualize the geographic origin of wound samples (figure 1). *S. aureus* positive wound swabs were phenotypically investigated towards methicillin-resistance based on susceptibility testing for oxacillin and cefoxitin (VITEK®2, bioMérieux, Germany). All MRSA were sent to the Institute of Microbiology and Epizootics, FU Berlin for genotypic characterization.

### Screening Procedure and Bacterial Characterization

All wound samples were streaked onto tryptic soy agar plates supplemented with 5% sheep blood (Becton Dickinson, Heidelberg, Germany) and the plates were investigated after 24 and 48 hours. Colonies suspected to be staphylococci were identified using the bioMérieux VITEK®2 system (Germany) according to the manufactureŕs instructions. Automated antimicrobial susceptibility testing of *S. aureus* was performed using the bioMerieux VITEK®2 system (Germany) according to the manufacturer’s instructions, including penicillin, ampicillin-sulbactam, oxacillin, cefoxitin, gentamicin, kanamycin, enrofloxacin, marbofloxacin, erythromycin, clindamycin, tetracycline, nitrofurantoin, chloramphenicol and trimethoprim- sulfamethoxazole, following the approved standards of the Clinical and Laboratory Standards Institute (CLSI) [21], [22].

MRSA isolates were stored in glycerol stocks at −80°C at the Institute of Microbiology and Epizootics (IMT). Species verification and detection of the *mec*A or *mec*C gene by PCR was performed for all MRSA as described before [23], [24]. Further, 121 MRSA were characterized by s*pa* typing [25]. Associations of *spa* types with corresponding clonal complexes (CC) were determined according to Ridom (http://spaserver.ridom.de/).

Multilocus sequence typing (MLST) was performed for one to seven representatives of each detected *spa* type as described elsewhere [26].

Detection of the genes encoding the Panton-Valentine leukocidin (PVL)-factor was carried out as described before [27].

## Results

**Figure 2:**
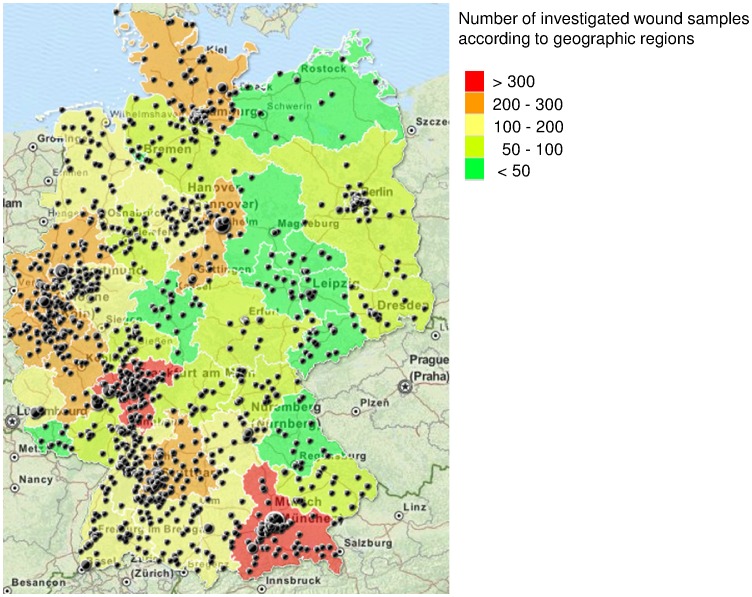
Overview of lineage-diversity among MRSA from dogs, cats and horses.

Within a 17-month sampling period, veterinarians from 15 German Federal States sent 3,479 swabs declared as “wound swabs” from dogs, 1,146 samples from cats and 604 samples from horses to Vet Med Labor GmbH (Ludwigsburg, Germany) (table 1). *S. aureus* was identified in 5.8% of canine, 12.2% of feline and 22.8% of equine samples, respectively. MRSA accounted for 62.7% of canine, 46.4% of feline and 41.3% of equine *S. aureus* isolates. In total, 121 MRSA strains obtained from dogs, 63 from cats and all isolates from horses were further characterized by *spa* typing while five of the canine and two feline MRSA were not available for the typing procedure. A detailed overview of all detected *spa* types and corresponding CCs is given in table 2. Most of the canine isolates belonged to clonal complexes CC22 (47.6%; n = 60) and CC5 (30.2%; n = 38), followed by CC398 (13.5%; n = 17), CC8 (4.0%; n = 5) and CC9 (0.8%; n = 1). Comparable results were obtained for feline strains with CC22 (49.2%; n = 32), CC5 (29.2%; n = 19), CC398 (7.7%; n = 5) and CC8 (9.2%; n = 6). In addition, a CC599-MRSA was identified in one feline sample. The majority of MRSA isolates of equine origin possessed *spa* types associated with CC398 (87.7%; n = 50). The remaining equine isolates belonged to CC8 (8.8%; n = 5) as well as CC5 and CC1 (each: one isolate) (figure 2).

**Table 1:** *S. aureus* proportion in wound samples from companion animals.

	total	%	dog	%	cat	%	horse	%
**wound swabs**	5,229	100	3,479	66.5	1,146	21.9	604	11.6
*S. aureus*	479	100	201	5.8	140	12.2	138	22.8
MSSA	231	48.2	75	2.2	75	6.5	81	13.4
MRSA	248	51.8	126	3.6	65	5.7	57	9.4

**Table 2:** *Spa* type frequency and predicted clonal complexes based on *spa* typing and MLST for representative MRSA from wound infections of canine, feline and equine origin in Germany.

		MRSA		
CC	*spa* type	dog (n = 121)	cat (n = 62)	horse (57)
**1**	t127	0	0	1
**5**	t002	1	1	0
	t003	31	17	1
	t045	4	1	0
	t1007	1	0	0
	t264	1	0	0
**8**	t008	2	6	0
	t009	1	0	4
	t024	1	0	0
	t12131	0	0	1
	t036	1	0	0
**9**	t1430	1	0	0
**22**	t016	0	1	0
	t020	0	4	0
	t022	11	6	0
	t032	43	17	0
	t3846	0	1	0
	t294	1	0	0
	t557	1	0	0
	t613	0	1	0
	t1292	1	0	0
	t747	2	0	0
	t7982	0	2	0
	t910	1	0	0
**599**	t278	0	1	0
**398**	t011	10	5	24
	t034	2	0	0
	t108	1	0	0
	t6867	4	0	24
	t10643	0	0	2

MLST analysis, performed for at least one representative isolate of each distinct *spa* type, revealed common sequence types (ST) for isolates of CC1 (ST1), CC5 (ST5, ST225), CC8 (ST8, ST254), CC9 (ST9) and CC398 (ST398). Representatives of CC22 showed sequence types ST22, ST1117 as well as two new sequence types (ST2743 and ST2745). The feline CC599-MRSA belonged to ST599. All MRSA were *mec*A-positive except for the ST599-MRSA, which yielded a positive signal for *mec*C [28]. Screening for PVL revealed one positive canine CC8-MRSA (t008) as well as one positive feline CC22-MRSA (t016).

Antimicrobial susceptibility testing was performed for all MRSA. A detailed overview of all phenotypic resistance profiles is given in table 3.

**Table 3:** Phenotypic resistance for 241 MRSA grouped according to their clonal complexes.

CC	CC22	CC5	CC398	CC8	CC1	CC9	ST599
total number	n = 92	n = 58	n = 72	n = 16	n = 1	n = 1	n = 1
GEN	3 (3.3%)	2 (3.4%)	57 (79.2%)	7 (43.8%)	0	0	0
KAN	3 (3.3%)	35 (60.3%)	58 (80.6%)	14 (87.5%)	1	0	0
ENR	90 (97.8%)	57 (98.2%)	28 (38.9%)	14 (87.5%)	0	1	0
MAR	90 (97.8%)	57 (98.2%)	30 (41.7%)	14 (87.5%)	0	1	0
ERY	32 (34.8%)	57 (98.2%)	7 (9.7%)	3 (18.8%)	1	1	0
CLI	34 (37.0%)	57 (98.2%)	8 (11.1%)	2 (12.5%)	1	1	0
TET	7 (7.6%)	1 (1.7%)	68 (94.4%)	7 (43.8%)	1	1	0
NIT	0	0	0	2 (12.5%)	0	0	0
CHL	1 (1.1%)	0	0	0	0	0	0
SXT	1 (1.1%)	1 (1.7%)	25 (34.7%)	6 (37.5%)	0	1	0

Number (n) and percentage (%) of resistant strains according to VITEK®2 system (bioMérieux, Germany).

Abbreviations: GEN: gentamicin, KAN: kanamycin, ENR: enrofloxacin, MAR: marbofloxacin, ERY: erythromycin, CLI: clindamycin, TET: tetracycline, NIT: nitrofurantoin, CHL: chloramphenicol and SXT: trimethoprim- sulfamethoxazole.

## Discussion

This survey describes for the first time the Germany-wide occurrence of MRSA and methicillin-susceptible *S. aureus* (MSSA) in wound samples of companion animal origin. The considerable high proportion of *S. aureus-*positive wound swabs from horses, cats and dogs clearly shows the prominent clinical importance of this pathogen in companion animal medicine. In view of MRSA being a zoonotic pathogen, proportions of 9.4% (horses), 5.7% (cats) and 3.6% (dogs) among wound swabs are alarming and provide a significant source for the spread in both veterinary clinics and the community.

The genotypic characterization of MRSA in this study provides comprehensive insights into the clonal background of MRSA in wound infection samples of companion animal origin (figure 2) and confirms the previous finding of CC22 and CC5 as predominant lineages in dogs and cats [14], [20] for Germany. The predominance of these lineages in dogs and cats is in accordance with findings in human medicine within Germany, where most wound infections are caused by CC22- and CC5-MRSA as well [29]. These findings lead to the suggestion that humans might be a likely infection source for dogs and cats. In addition, dogs and cats should be considered as (re-)infection and/or colonization source for humans as well.

However, a recent study by Holden et al. demonstrated the genetic changes that accompanied the emergence of EMRSA-15, a typical hospital associated CC22-MRSA. A series of gene acquisition events and mutations seemed to promote the success of this clone in (human) hospitals. Accordingly, further comparative genomic studies on MRSA obtained from veterinary and human health care settings are warranted to evaluate factors influencing success in veterinary environments (e.g. prescription of certain antibiotics, acquisition of phages encoding virulence-enhancing factors) [30].

Reports about LA-MRSA in pet animals [15], [31], [32] raised the question whether these results represent incidental findings from screening studies in companion animals [33]. As shown in this study, the frequent finding of LA-MRSA CC398 among canine and feline MRSA isolates demonstrates that CC398-MRSA is an important lineage for canine and feline infections in Germany at present. Further investigation is needed to determine whether this genotype has the capacity to replace other common MRSA-lineages in dogs and cats. However, the characterization of different CC398 clades among humans in comparison to livestock already demonstrated the adaptability of this genetic lineage [34]. Based on this knowledge and the regular appearance of CC398-MRSA in pet animals, dogs and cats should be considered as a potential source for human CC398-MRSA colonization and infection, especially if contact to livestock or horses can be excluded.

The CC398 proportion among equine MRSA identified in this study is in concordance with previous reports of high CC398-MRSA infection- and colonization rates in horses [7], [18] and gives evidence that MRSA of CC8 are almost completely replaced by CC398 in equine wound infections within Germany. The predominance of CC398-MRSA in wound swabs of horse origin leads to the assumption that this lineage might have developed some mechanisms for adaptation to the equine host.

Host adaptation has been described for several *S. aureus-*lineages in the past. The emergence of a poultry-specific ST5-clade for example was probably caused by a single human-to-poultry host jump. Further acquisition of mobile genetic elements (MGE) from avian specific lineages and the inactivation of several proteins with importance in human infection led to the occurrence of a poultry-specific clade [35]. Another example was given for CC97-*S. aureus,* a lineage that has been predominantly identified in samples from ruminants in the past. Only recently, CC97-MRSA were identified as a cause of human infections as well. The emergence of two new CC97 clones in the human population seems to be associated with the acquisition of MGE which encode factors that enhance the capacity to adapt to the human host [36].

In accordance with findings for CC5- and CC97-*S. aureus,* different studies revealed the capacity of CC398 to adapt to specific hosts including humans and livestock. The recent analysis of 89 diverse CC398-*S. aureus* from Price et al. strongly suggests an initial jump from humans to livestock, followed by adaptive processes like the loss of phage-carried human-associated virulence genes and the acquisition of tetracycline and methicillin resistance [37]. Besides, a recent study showed the emergence of an animal-independent ST398-MSSA with relevance in human infections in northern Manhattan. In addition to a different subset of MGE, these strains varied according to the set of intact adhesins, resulting in enhanced adhesion to human keratinocytes and keratin and demonstrated the adaptive capacity of ST398 strains to humans [38]. Further investigation is required to see if CC398-MRSA of equine origin harbour similar adaptive features.

Only one of the 243 MRSA yielded a positive signal for the *mec*A-homologue *mec*C, indicating a sporadic occurrence of *mec*C-MRSA in companion animals in Germany, as previously reported [28]. A rather sporadic occurrence of PVL-carriage among companion animal MRSA strains (two positives) is in accordance with previous findings [39], [40].

Phenotypic resistance profiles of all MRSA revealed a high number of multi-resistant strains, in particular for CC5- and CC398-MRSA, highlighting the limited therapeutic options to treat most of these MRSA infections (table 3). Resistance profiles from isolates of human origin associated with epidemic MRSA lineages like CC22, CC5 and CC8 within German hospitals were regularly reported by the national reference laboratory Robert Koch-Institute (RKI) [29]. For ST22, the “Barnim epidemic strain” (*spa* types t005, t002 and t032), resistance against fluoroquinolones (ciprofloxacin, moxifloxacin) was frequently observed. While care should be taken before comparing study results (CC22-MRSA reported on here comprise more than these three *spa* types), the frequent resistance (97.8%) against fluoroquinolones (enrofloxacin, marbofloxacin) is also present in CC22-MRSA of companion animal origin. CC5-MRSA, human as well as companion animal strains, seem to express resistance against fluoroquinolones, clindamycin and erythromycin on a regular basis. For CC8-MRSA, a diverse phenotypic resistance pattern was observed. These results are in accordance with findings for CC8-MRSA of human origin [41].

The study was biased to some degree due to inhomogeneity of the case definition “wound infection” which was dependent on veterinarians who sent their samples to Vet Med Labor GmbH (Ludwigsburg, Germany). This survey focused on wound infections which have been sent to a diagnostic lab for microbiological examination but does not take empirically treated wound infections into account. Thus, calculated MRSA proportions might not reflect the MRSA and MSSA rates in the general companion animal population.

Another limitation is the lack of information for seven strains (five canine and two feline MRSA), which were not available for genotypic characterization.

## Conclusion

This study provides evidence for the importance and impact of *S. aureus* in general, and MRSA in particular, as a cause of wound infections in dogs, cats and horses. The emergence of CC398-MRSA as predominant genotype in equine wound infections demonstrates the adaptive capacity of this lineage to other hosts than livestock and humans. Since CC398-MRSA can be also regularly identified in samples from dogs and cats, each of these animals should be considered as potential infection and/or colonization source for humans as well. Our findings clearly demonstrate the need for on-going screening studies to gain information about the replacement and emergence of certain genetic lineages among these animals. Further investigation including whole genome sequencing, phenotypic appearance and functional studies are needed to unveil factors and structures influencing the host adaptation processes like it has been successfully demonstrated for adaptation of CC398 to humans [38] and a CC5-clade to poultry [35]. Further attempts to reduce the total burden of MRSA, in each field afflicted with this pathogen, are clearly needed. Transmission of MRSA between different hosts (humans, animals) as well as between different ecological niches (hospital, community, veterinarian hospitals) should be regarded as a major “One Health” problem.
