# Correction: Alarming Proportions of Methicillin-Resistant *Staphylococcus aureus* (MRSA) in Wound Samples from Companion Animals, Germany 2010–2012

**DOI:** 10.1371/journal.pone.0096965

**Published:** 2014-04-29

**Authors:** The PLOS ONE Staff

The images for Figure 1 and Figure 2 are reversed. Please view the correct images and legends for Figure 1 and Figure 2 here.

**Figure 1:**
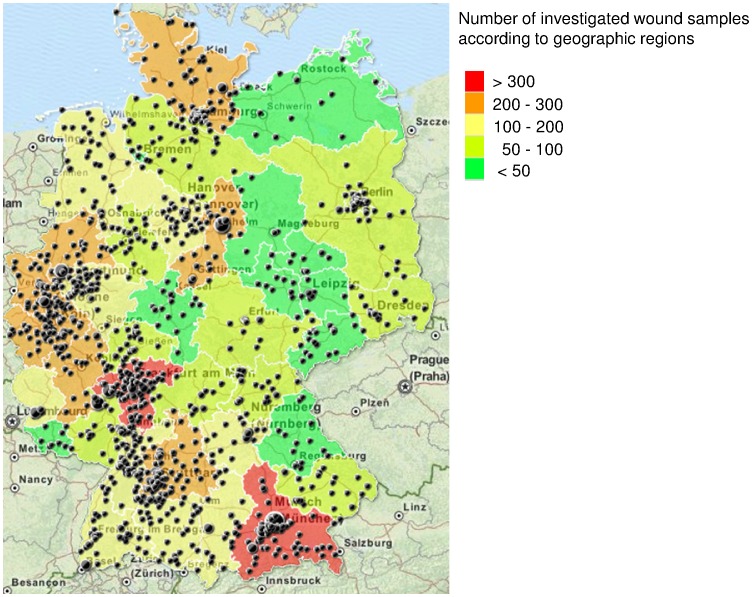
Sample origin. Figure 1 shows the Germany-wide origin of the 5,229 wound swabs from dogs, cats and horses. Areas are shaped in color with regard to the sample frequency. Black dots represent the sample origin with regard to the postal code. The dot size displays the submission frequency of each veterinary practice/clinic.

**Figure 2:**
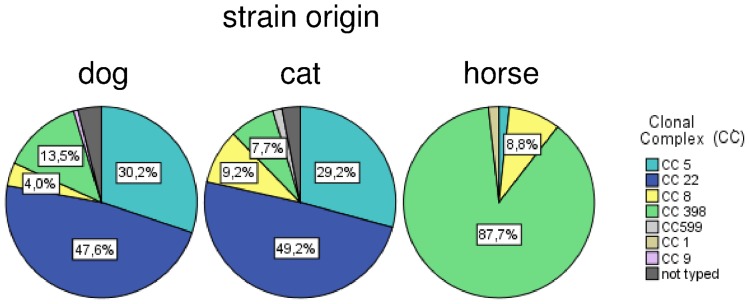
Overview of lineage-diversity among MRSA from dogs, cats and horses.
